# 423. Development and Validation of a Predictive Model of New-Onset Deep Vein Thrombosis/Pulmonary Embolism Among Hospitalized COVID-19 Patients: A Retrospective Cohort Study

**DOI:** 10.1093/ofid/ofad500.493

**Published:** 2023-11-27

**Authors:** Qingqing Meng, Guillermo Rodriguez-Nava, Goar Egoryan, Armen Kishmiryan

**Affiliations:** Ascension Saint Francis Hospital, Evanston, Illinois; Stanford University School of Medicine, Palo Alto, California; Ascension Health Saint Francis Hospital, Evanston, Illinois; Ascension Health, Evanston, Illinois

## Abstract

**Background:**

COVID-19 can increase the risk of thromboembolism events, such as deep vein thrombosis (DVT) and pulmonary embolism (PE). This study aimed to develop a risk prediction model for new-onset DVT/PE in hospitalized COVID-19 patients based on baseline characteristics, major complaints, and lab values.

**Methods:**

Retrospective data from 671 hospitalized COVID-19 patients were collected between March and October 2020. After excluding missing values, 241 cases were included in the final analysis (Table 1). Lasso regression was used to select related variables, and a prediction model was built using logistic regression (Figure 1). A nomogram was established based on multivariate analysis, and the model was validated using the bootstrap method.

**Figure:**
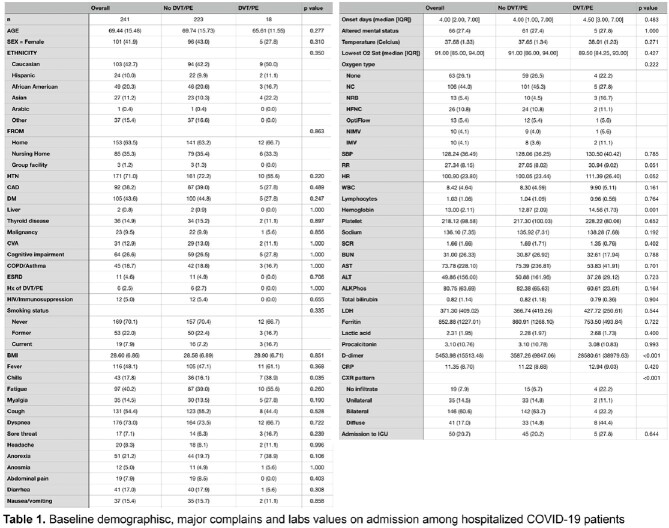

**Figure:**
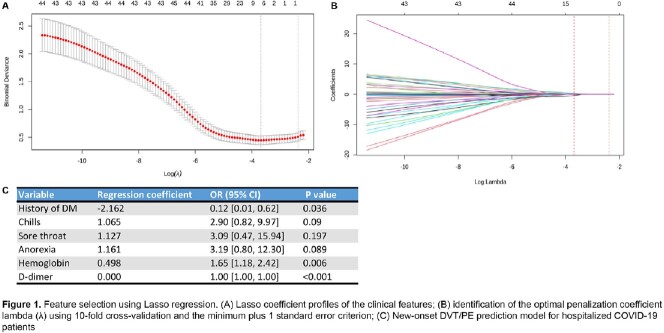

**Results:**

Among the 241 hospitalized patients with COVID-19 infection, 18 (7.5%) developed new-onset DVT/PE during hospitalization. The final prediction model included the history of diabetes mellitus, chills, sore throat, anorexia, hemoglobin, and D-dimer levels on admission. A predictive nomogram model for new-onset DVT/PE based on these variables was built (Figure 2). Our prediction model showed good predictive performance with an area under the curve (AUC) of 0.89 and a bootstrap-corrected AUC of 0.82 (95% CI: 0.767-0.873) (Figure 3). The calibration plots demonstrated good agreement between the estimated probability and the actual observation (Figure 4A). Decision curve analysis shows the nomogram is clinically useful (Figure 4B).

**Figure:**
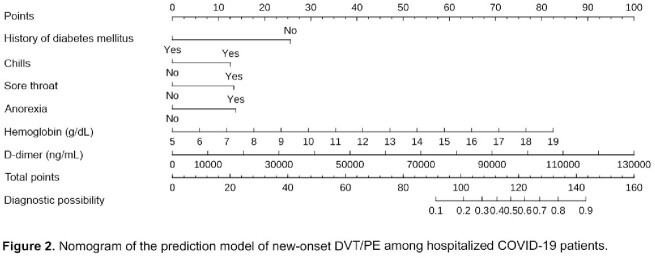

**Figure:**
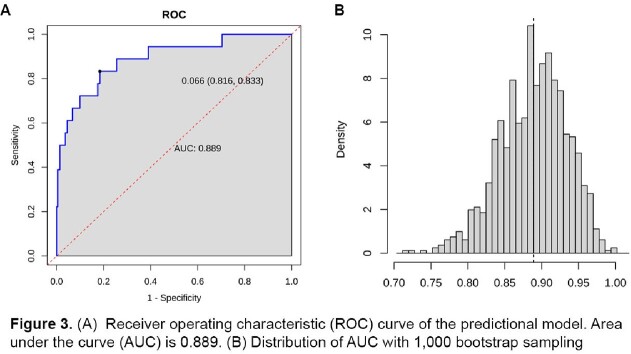

**Figure:**
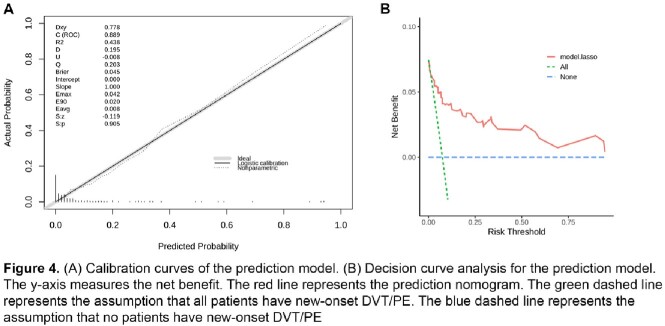

**Conclusion:**

The developed model for new-onset DVT/PE in hospitalized COVID-19 patients based on baseline characteristics, major complaints, and lab values had good discrimination and calibration. It may help identify high-risk patients who require close monitoring and prophylactic anticoagulation. Future studies are needed to validate the model in different populations and settings.

**Disclosures:**

**All Authors**: No reported disclosures

